# Genetic diversity of *Salmonella enterica* isolated over 13 years from raw California almonds and from an almond orchard

**DOI:** 10.1371/journal.pone.0291109

**Published:** 2023-09-07

**Authors:** Anne-laure Moyne, Opeyemi U. Lawal, Jeff Gauthier, Irena Kukavica-Ibrulj, Marianne Potvin, Lawrence Goodridge, Roger C. Levesque, Linda J. Harris

**Affiliations:** 1 Department of Food Science and Technology, University of California, Davis, California, United States of America; 2 Western Center for Food Safety, University of California, Davis, California, United States of America; 3 Canadian Research Institute for Food Safety, Department of Food Science, University of Guelph, Guelph, Ontario, Canada; 4 Institut de biologie intégrative et des systèmes (IBIS), Faculté de médecine, Université Laval, Québec, Québec, Canada; 5 Food Safety and Quality Program, Department of Food Science and Agricultural Chemistry, McGill University, Sainte Anne de Bellevue, Quebec, Canada; Nitte University, INDIA

## Abstract

A comparative genomic analysis was conducted for 171 *Salmonella* isolates recovered from raw inshell almonds and raw almond kernels between 2001 and 2013 and for 30 *Salmonella* Enteritidis phage type (PT) 30 isolates recovered between 2001 and 2006 from a 2001 salmonellosis outbreak-associated almond orchard. Whole genome sequencing was used to measure the genetic distance among isolates by single nucleotide polymorphism (SNP) analyses and to predict the presence of plasmid DNA and of antimicrobial resistance (AMR) and virulence genes. Isolates were classified by serovars with Parsnp, a fast core-genome multi aligner, before being analyzed with the CFSAN SNP Pipeline (U.S. Food and Drug Administration Center for Food Safety and Applied Nutrition). Genetically similar (≤18 SNPs) *Salmonella* isolates were identified among several serovars isolated years apart. Almond isolates of *Salmonella* Montevideo (2001 to 2013) and *Salmonella* Newport (2003 to 2010) differed by ≤9 SNPs. *Salmonella* Enteritidis PT 30 isolated between 2001 and 2013 from survey, orchard, outbreak, and clinical samples differed by ≤18 SNPs. One to seven plasmids were found in 106 (62%) of the *Salmonella* isolates. Of the 27 plasmid families that were identified, IncFII and IncFIB plasmids were the most predominant. AMR genes were identified in 16 (9%) of the survey isolates and were plasmid encoded in 11 of 16 cases; 12 isolates (7%) had putative resistance to at least one antibiotic in three or more drug classes. A total of 303 virulence genes were detected among the assembled genomes; a plasmid that harbored a combination of *pef*, *rck*, and *spv* virulence genes was identified in 23% of the isolates. These data provide evidence of long-term survival (years) of *Salmonella* in agricultural environments.

## Introduction

From 2001 to 2006, *Salmonella enterica* serovar Enteritidis was implicated in three outbreaks linked to raw almond consumption. Epidemiologic and traceback investigations of a 2000 to 2001 (denoted 2001) salmonellosis outbreak in Canada and in the United States identified a rare phage type (PT) of *Salmonella* Enteritidis, PT 30, from clinical samples, raw almonds sampled at retail, environmental (swab) samples at an almond processor and a huller-sheller facility, and environmental drag swabs obtained from multiple orchards in California [1]. *Salmonella* Enteritidis PT 30 was recovered in 2001 (three of six samples collected) [1]) and, in a subsequent study, at least two times in each year between 2002 and 2006 from environmental drag swabs collected in one of these orchards (eight to 96 samples collected each year) [2]. Raw almonds were epidemiologically linked to clinical cases of *Salmonella* Enteritidis PT 30 reported in Sweden in 2005 to 2006 (denoted 2006) [3] but *Salmonella* was not isolated from the implicated California almonds. Between 2003 and 2004 (denoted 2004), another rare phage type, *Salmonella* Enteritidis PT 9c, was linked to consumption of raw California almonds in the United States [4].

Excluding the farms linked to the 2001 outbreak, the prevalence and levels of *Salmonella* were determined in a multi-year survey of raw almond kernels. Samples of individual lots of raw almond kernels were collected as they were received by handlers (processors) located throughout the almond-growing regions of California from 2001 to 2007, and in 2010 and 2013 [5–7]. Inshell almonds were included in the survey in 2006 and 2007 [6]. The prevalence of *Salmonella* in 14,949 lots of raw almond kernels was 0.98% ± 0.29% over the 9 years (146 positive 100-g samples) [7]. Levels of *Salmonella* were estimated for 118 of the positive lots; mean and median levels of *Salmonella* were 1.14 ± 1.69 and 0.79 most probable number (MPN)/100 g, respectively, with single lots at 9.25 and 15.4 MPN/100 g [7]. Of the small number of raw inshell almond lots (455) evaluated in 2006 and 2007, 1.5% (seven 100-g subsamples) were positive [6]. Using classical serological nomenclature, the *Salmonella* isolates retrieved from these surveys were serotyped into 45 different serovars, including *Salmonella* Enteritidis PT 30 and PT 9c [5, 6].

Pulsed-field gel electrophoresis (PFGE), multilocus variable-number tandem repeat analysis (MLVA), and comparative genomic indexing (CGI) were applied to *Salmonella* Enteritidis strains associated with the 2001, 2004, and 2006 almond outbreaks, including clinical and environmental isolates [8]. The *Salmonella* Enteritidis PT 30 and PT 9c strains could be separated from each other and from other *Salmonella* Enteritidis phage types based on DNA enzyme restriction profiles, MLVA types, and genes identified by CGI. However, neither PFGE nor MVLA could discriminate among the *Salmonella* Enteritidis PT 30 isolates associated with the 2001 and 2006 almond-associated outbreaks. *Salmonella* Enteritidis PT 30 almond-associated outbreak strains could not be distinguished from epidemiologically unrelated *Salmonella* Enteritidis PT 30 clinical strains included in the study [8]. Among *Salmonella* Enteritidis PT 30 strains with identical genotypes, metabolic analyses with Biolog revealed differences between clinical and environmental isolates [8], indicating the discriminatory limit of the then current genotyping methods. Environmental *Salmonella* Enteritidis PT 30 isolates, collected between 2001 and 2006 from one of the 2001 outbreak-associated orchards, clustered in two groups based on the separation by PFGE of their XbaI-digested DNA [2].

Whole genome sequencing (WGS) has replaced PFGE for investigating foodborne outbreaks because of its higher resolution, and this methodology has been incorporated into routine public health surveillance since 2014 [9, 10]. Clonality of pathogen strains determined by WGS analyses can provide information about contamination during food production and distribution [11]. Different workflows have been developed to assess levels of genetic relatedness of foodborne pathogens by the National Center for Biotechnology Information (NCBI) with the Pathogen Detection Pipeline (https://www.ncbi.nlm.nih.gov/pathogens/), the U.S. Food and Drug Administration with the Center for Food Safety and Applied Nutrition CFSAN SNP Pipeline [12], and the U.S. Centers for Disease Control and Prevention with Lyve-SET [13], based on the specific needs of the respective agencies. The CFSAN SNP Pipeline creates high quality SNP matrices from WGS data that allow connection between clinical isolates to food or environmental isolates based on their evolutionary relationship [12]. Resident pathogen strains in facilities will have closely related WGS profiles, whereas transient pathogen strains will have unique or unrelated WGS profiles [11, 14].

The objective of the present study was a comparative genomic analysis of 171 *Salmonella* (45 serovars) isolated from raw inshell almonds and almond kernels in 9 years of surveys conducted between 2001 and 2013; 30 *Salmonella* Enteritidis PT 30 isolates recovered between 2001 and 2006 from a 2001 outbreak-associated almond orchard were included in the analysis. The CFSAN SNP Pipeline was selected to measure the genetic distances among the isolates. WGS was used to predict antimicrobial resistance (AMR), virulence genes, and presence of plasmid DNA.

## Materials and methods

### Isolate selection

Isolates were retrieved from enrichment of raw almond kernels and inshell almonds (survey isolates), and from swabs that were dragged across the floor of one of the 2001 outbreak-associated orchards (orchard isolates). A total of 15,505 ∼1-kg samples from different lots of raw almond kernels (14,949) and inshell almonds (455) were collected upon receipt at several almond processors located throughout California from the 2001–2005 [5], 2006–2007 [6], 2010 ([15]; present study), and 2013 ([7]; present study) harvests, as described previously. All samples were coded to keep the identities of the processors confidential; the geographic origins of the samples were unknown. The Safe Food Alliance, Kingsburg, CA (formerly American Council for Food Safety and Quality, Fresno, CA) analyzed a 100-g subsample from each lot of almonds by enriching for the presence of *Salmonella* [5]. In addition, the MPN of *Salmonella* was determined for 118 samples by enriching one or more additional samples weighing from ∼0.25 to 100 g. *Salmonella* isolates were stored at −80°C.

A total of 171 *Salmonella* survey isolates were selected for the present study: for each individual positive almond lot, a single *Salmonella* isolate retrieved from the initial 100-g subsample (153 positive samples, 148 isolates; three, one, and one isolate from 2001, 2002, and 2003, respectively, were lost), any isolates recovered from MPN or secondary enrichments that differed from the initial serotype (12), and unique isolates recovered from secondary enrichments of initially negative samples (11) (Tables 1 and S1 in S1 File). Traditional serotyping was done for each banked *Salmonella* by the California Animal Health and Food Safety Laboratory System (Davis, CA), and phage typing for *Salmonella* Enteritidis isolates was done by the National Veterinary Services Laboratory (Ames, IA) (Table 1). *Salmonella* Enteritidis PT 9c LJH1024 (obtained from Robert Mandrell, U.S. Department of Agriculture, Agricultural Research Service; RM4635 or G04-101 [8], raw almond isolate from the 2004 outbreak) and *Salmonella* Enteritidis PT 30 LJH0608 (raw almond isolate from the 2001 outbreak that was deposited to the American Type Culture Collection as ATCC BAA-1045) were also included in some of the analyses (S2 Table in S1 File).

**Table 1:** Results of the WGS serotyping and number of plasmids for *Salmonella* survey isolates retrieved from raw almonds.

Strain designation	Isolation year	Accession number	WGS serotyping		Traditional serotyping	Number of plasmids
			Subspecies	WGS serotyping		
LJH0651	2001	SRR23719048	*enterica*	Brandenburg	Brandenburg	0
LJH0652	2001	SRR23719047	*enterica*	Thompson	Thompson	0
LJH0653	2001	SRR23718942	*enterica*	Montevideo	Montevideo	4
LJH0654	2001	SRR23718931	*enterica*	Montevideo	Montevideo	0
LJH0655	2001	SRR23718920	*enterica*	Brandenburg	Brandenburg	0
LJH0656	2001	SRR23718909	*enterica*	Newport	Newport	2
LJH0657	2001	SRR23718898	*enterica*	Montevideo	Montevideo	0
LJH0658	2001	SRR23718887	*enterica*	Give	Nancy (Nchanga)	3
LJH0659	2001	SRR23719007	*enterica*	Montevideo	Montevideo	1
LJH0666	2002	SRR23718996	*enterica*	Typhimurium	Typhimurium	0
LJH0667	2002	SRR23719046	*enterica*	Senftenberg	Senftenberg	4
LJH0668	2002	SRR23718976	*enterica*	Give	Give	4
LJH0669	2002	SRR23718965	*enterica*	Typhimurium	Typhimurium	0
LJH0687	2002	SRR23718954	*enterica*	1,4,[5],12:i:-	1,4,[5],12:i:-	1
LJH0690	2002	SRR23719038	*enterica*	Oranienburg	Oranienburg	1
LJH0691	2002	SRR23719027	*enterica*	Oranienburg	Oranienburg	1
LJH0692	2002	SRR23719016	*enterica*	Worthington	Worthington	1
LJH0693	2002	SRR23718946	*enterica*	Heidelberg	Worthington	2
LJH0694	2002	SRR23718944	*enterica*	Oranienburg	Muenchen	1
LJH0695	2002	SRR23718943	*enterica*	Newport	Heidelberg	1
LJH0696	2002	SRR23718941	*enterica*	Thompson	Agona	0
LJH0697	2002	SRR23718940	*enterica*	Newport	Newport	1
LJH0698	2002	SRR23718939	*enterica*	Manhattan	Agona	1
LJH0713	2002	SRR23718938	*enterica*	Senftenberg	Senftenberg	0
LJH0714	2002	SRR23718937	*enterica*	Lomalinda	Lomalinda	1
LJH0715	2002	SRR23718936	*enterica*	Tennessee	Tennessee	1
LJH0716	2002	SRR23718935	*enterica*	Braenderup	Braenderup	2
LJH0717	2002	SRR23718934	*enterica*	Typhimurium	Typhimurium	0
LJH0718	2002	SRR23718933	*enterica*	Schwarzengrund	Schwarzengrund	3
LJH0719	2002	SRR23718932	*enterica*	Montevideo	Montevideo	1
LJH0720	2002	SRR23718930	*enterica*	Anatum	Anatum	1
LJH0721	2002	SRR23718929	*enterica*	Tennessee	Tennessee	0
LJH0722	2002	SRR23718928	*enterica*	Infantis	Infantis	0
LJH0724	2002	SRR23718927	*enterica*	Zerifin	Zerifin	4
LJH0725	2003	SRR23718926	*enterica*	Horsham	Brandenburg	0
LJH0726	2003	SRR23718925	*enterica*	Indiana	Indiana	7
LJH0738	2003	SRR23718924	*enterica*	1,4,[5],12:i:-	Typhimurium	1
LJH0739	2003	SRR23718923	*enterica*	Thompson	Thompson	0
LJH0740	2003	SRR23718922	*enterica*	Thompson	Thompson	1
LJH0741	2003	SRR23718921	*enterica*	Thompson	Thompson	1
LJH0742	2003	SRR23718919	*enterica*	Sandiego	Sandiego	1
LJH0751	2003	SRR23718918	*enterica*	Newport	Newport	1
LJH0752	2003	SRR23718917	*enterica*	Oranienburg	Othmarschen	1
LJH0753	2003	SRR23718916	*enterica*	Istanbul	Istanbul	3
LJH0754	2003	SRR23718915	*enterica*	Muenchen	Newport	1
LJH0759	2003	SRR23718914	*enterica*	Montevideo	Montevideo	0
LJH0760	2003	SRR23718913	*enterica*	Montevideo	Montevideo	1
LJH0761	2003	SRR23718912	*enterica*	Typhimurium	Typhimurium	1
LJH0762	2003	SRR23718911	*enterica*	Enteritidis	Enteritidis	1
LJH0783	2004	SRR23718910	*enterica*	Liverpool	Liverpool	0
LJH0784	2004	SRR23718908	*enterica*	Braenderup	Braenderup	1
LJH0787	2004	SRR23718907	*enterica*	Anatum	Anatum	3
LJH0788	2004	SRR23718906	*enterica*	Typhimurium	Typhimurium var. Copenhagen	1
LJH0789	2004	SRR23718905	*enterica*	Montevideo	Montevideo	0
LJH0790	2004	SRR23718904	*enterica*	Horsham	Horsham	0
LJH0791	2004	SRR23718903	*enterica*	Thompson	Thompson	1
LJH0792	2004	SRR23718902	*enterica*	Thompson	Thompson	1
LJH0793	2004	SRR23718901	*enterica*	Thompson	Thompson	1
LJH0794	2004	SRR23718900	*enterica*	Thompson	Thompson	1
LJH1011	2004	SRR23718899	*enterica*	Senftenberg	Senftenberg	0
LJH1012	2004	SRR23718897	*enterica*	Anatum	Anatum	2
LJH1013	2005	SRR23718896	*enterica*	Newport	Saintpaul	0
LJH1019	2005	SRR23718895	*enterica*	Give	Give	2
LJH1020	2005	SRR23718894	*enterica*	Montevideo	Montevideo	1
LJH1021	2005	SRR23718893	*enterica*	Heidelberg	Heidelberg	2
LJH1022	2005	SRR23718892	*enterica*	Mbandaka	Mbandaka	0
LJH1023	2005	SRR23718891	*enterica*	Enteritidis	Enteritidis	3
LJH1025	2005	SRR23718890	*enterica*	Typhimurium	Typhimurium	0
LJH1026	2005	SRR23718889	*enterica*	Manhattan	Untypeable	0
LJH1027	2005	SRR23718888	*enterica*	Muenchen	Muenchen	0
LJH1028	2005	SRR23718886	*enterica*	Enteritidis	Enteritidis	1
LJH1029	2005	SRR23718885	*enterica*	Enteritidis	Untypeable	1
LJH1030	2005	SRR23718884	*enterica*	Tennessee	Tennessee	0
LJH1043	2005	SRR23718883	*enterica*	Typhimurium	Typhimurium var. Copenhagen	1
LJH1044	2005	SRR23718882	*enterica*	Kentucky	Kentucky	2
LJH1045	2005	SRR23718881	*enterica*	Montevideo	Montevideo	0
LJH1046	2005	SRR23718880	*enterica*	Enteritidis	Enteritidis	1
LJH1047	2005	SRR23718879	*enterica*	Enteritidis	Enteritidis	0
LJH1048	2005	SRR23718878	*enterica*	Enteritidis	Enteritidis	1
LJH1049	2005	SRR23719008	*enterica*	Enteritidis	Enteritidis	1
LJH1052	2005	SRR23719006	*enterica*	Duisburg	Duisburg	3
LJH1054	2006	SRR23719005	*enterica*	Typhimurium	Typhimurium	1
LJH1055	2006	SRR23719004	*enterica*	Typhimurium	Typhimurium	1
LJH1056	2006	SRR23719003	*enterica*	Typhimurium	Typhimurium	1
LJH1058	2006	SRR23719002	*enterica*	Muenchen	Muenchen	0
LJH1059	2006	SRR23719001	*enterica*	Enteritidis	Enteritidis	1
LJH1063	2006	SRR23719000	*enterica*	Anatum	Anatum	1
LJH1067	2006	SRR23718999	*diarizonae* (IIIb)		III 50:k:-	0
LJH1068	2006	SRR23718998	*enterica*	Newport	Newport	0
LJH1070	2006	SRR23718997	*enterica*	Heidelberg	Heidelberg	2
LJH1071	2006	SRR23718995	*enterica*	Give	Give	2
LJH1076	2006	SRR23718994	*enterica*	Muenchen	Muenchen	0
LJH1080	2006	SRR23718993	*enterica*	Muenchen	Muenchen	0
LJH1082	2006	SRR23718992	*enterica*	1,4,[5],12:i:-	1,4,[5],12:i:-	1
LJH1083	2006	SRR23718991	*enterica*	Muenchen	Muenchen	0
LJH1084	2006	SRR23718990	*enterica*	Newport	Newport	1
LJH1085	2006	SRR23718989	*enterica*	Newport	Muenchen	0
LJH1087	2006	SRR23718988	*enterica*	Muenchen	Muenchen	0
LJH1088	2006	SRR23718987	*enterica*	Muenchen	Newport	0
LJH1089	2006	SRR23718985	*enterica*	Horsham	Horsham	0
LJH1090	2006	SRR23719045	*enterica*	Muenchen	Muenchen	0
LJH1094	2006	SRR23718986	*enterica*	Montevideo	Montevideo	0
LJH1095	2006	SRR23718984	*enterica*	1,4,[5],12:i:-	1,4,[5],12:i:-	1
LJH1096	2006	SRR23718983	*enterica*	Enteritidis	Enteritidis	2
LJH1097	2006	SRR23718982	*enterica*	Oranienburg	Oranienburg	1
LJH1098	2006	SRR23718981	*enterica*	1,4,[5],12:i:-	1,4,[5],12:i:-	1
LJH1099	2006	SRR23718980	*enterica*	Meleagridis	Meleagridis	0
LJH1100	2006	SRR23718979	*enterica*	Agona	Agona	1
LJH1101	2006	SRR23718978	*enterica*	Muenchen	Muenchen	0
LJH1102	2006	SRR23718977	*enterica*	Montevideo	Montevideo	0
LJH1103	2006	SRR23718975	*enterica*	Enteritidis	Enteritidis	1
LJH1104	2006	SRR23718974	*enterica*	Enteritidis	Enteritidis	1
LJH1105	2006	SRR23718973	*enterica*	Give	Give	1
LJH1106	2006	SRR23718972	*enterica*	Agona	Agona	2
LJH1107	2006	SRR23718971	*enterica*	Newport	Newport	2
LJH1108	2006	SRR23718970	*enterica*	Muenchen	Muenchen	0
LJH1109	2006	SRR23718969	*enterica*	Enteritidis	Enteritidis	4
LJH1133	2007	SRR23718968	*enterica*	Newport	Newport	0
LJH1134	2007	SRR23718967	*enterica*	Cerro	Cerro	0
LJH1135	2007	SRR23718966	*enterica*	Muenchen	Muenchen	0
LJH1136	2007	SRR23718964	*enterica*	Cerro	Cerro	0
LJH1137	2007	SRR23718963	*enterica*	Manhattan	Manhattan	1
LJH1138	2007	SRR23718962	*enterica*	Newport	Newport	2
LJH1139	2007	SRR23718961	*enterica*	Thompson	Thompson	0
LJH1140	2007	SRR23718960	*enterica*	Irumu	Irumu	0
LJH1141	2007	SRR23718959	*enterica*	Typhimurium	Typhimurium	2
LJH1142	2007	SRR23718958	*arizonae* (IIIa)		IIIa 18:z32:-	0
LJH1143	2007	SRR23718957	*enterica*	Oranienburg	Othmarschen	1
LJH1144	2007	SRR23718956	*enterica*	Typhimurium	I 4,12:i:-	3
LJH1145	2007	SRR23718955	*enterica*	Brandenburg	Brandenburg	2
LJH1146	2007	SRR23718953	*enterica*	Thompson	Thompson	0
LJH1147	2007	SRR23718952	*enterica*	Give	Bredeney	1
LJH1148	2007	SRR23718951	*enterica*	Newport	Newport	0
LJH1149	2007	SRR23718950	*enterica*	Cerro	Cerro	0
LJH1150	2007	SRR23719044	*enterica*	Senftenberg	Senftenberg	0
LJH1151	2007	SRR23719043	*enterica*	Muenchen	Untypeable	1
LJH1154	2007	SRR23719042	*enterica*	Senftenberg	Senftenberg	0
LJH1248-1	2010	SRR23719041	*enterica*	Newport	Newport	1
LJH1249-1	2010	SRR23719040	*enterica*	Give	Give	1
LJH1250-1	2010	SRR23719039	*enterica*	Infantis	Infantis	0
LJH1251-1	2010	SRR23719037	*enterica*	Give	Give	1
LJH1252-1	2010	SRR23719036	*enterica*	Infantis	Infantis	0
LJH1266-1	2010	SRR23719035	*arizonae* (IIIa)		II:17:g,t:-	0
LJH1267-1	2010	SRR23719034	*enterica*	Mbandaka	Mbandaka	0
LJH1268-1	2010	SRR23719033	*enterica*	Infantis	Infantis	0
LJH1269-1	2010	SRR23719032	*enterica*	Duisburg	Duisburg	1
LJH1270-1	2010	SRR23719031	*enterica*	Heidelberg	Heidelberg	2
LJH1271-1	2010	SRR23719030	*enterica*	Infantis	Infantis	0
LJH1272-1	2010	SRR23719029	*enterica*	Enteritidis	Enteritidis	1
LJH1273-1	2010	SRR23719028	*enterica*	Newport	Newport	1
LJH1276-1	2010	SRR23719026	*enterica*	Oranienburg	Othmarschen	1
LJH1277-1	2010	SRR23719025	*enterica*	Heidelberg	Heidelberg	2
LJH1278-1	2010	SRR23719024	*enterica*	Newport	Newport	1
LJH1618-1	2013	SRR23719023	*enterica*	1,4,[5],12:i:-	1,4,[5],12:i:-	1
LJH1619-1	2013	SRR23719022	*enterica*	Muenchen	I 6:8:d:z6	1
LJH1620-1	2013	SRR23719021	*enterica*	Muenchen	Untypeable	1
LJH1622-1	2013	SRR23719020	*enterica*	Heidelberg	Heidelberg	1
LJH1623-1	2013	SRR23719019	*enterica*	Montevideo	Montevideo	0
LJH1624-1	2013	SRR23719018	*diarizonae* (IIIb)	P:k:z35	Untypeable	0
LJH1628-1	2013	SRR23719017	*enterica*	Montevideo	Montevideo	1
LJH1629-1	2013	SRR23719015	*enterica*	Give	Give	1
LJH1630-1	2013	SRR23719014	*enterica*	Cerro	Cerro	2
LJH1631-1	2013	SRR23719013	*enterica*	Give	Give	2
LJH1633-1	2013	SRR23719012	*enterica*	Enteritidis	Enteritidis	2
LJH1660-1	2013	SRR23719011	*arizonae* (IIIa)		IIIa 41:z23: -	0
LJH1661-1	2013	SRR23719010	*enterica*	Muenchen	Muenchen	0
LJH1662-1	2013	SRR23719009	*enterica*	1,4,[5],12:i:-	Typhimurium	1
LJH1664-1	2013	SRR23718949	*diarizonae* (IIIb)		IIIb 50:r:z	0
LJH1665	2013	SRR23718948	*enterica*	1,4,[5],12:i:-	1,4,[5],12:i:-	1
LJH1673	2013	SRR23718947	*enterica*	Enteritidis	Enteritidis	2
LJH1676	2013	SRR23718945	*enterica*	1,4,[5],12:i:-	Typhimurium	1

Several 2001 outbreak-associated almond orchards sampled by investigators during the 2001 outbreak investigation were positive for *Salmonella* Enteritidis PT 30 [1]. One of these orchards was sampled every year from 2001 [1] through 2006 [2]. Briefly, sterile gauze swabs attached to a string and soaked in full-strength evaporated skim milk were pulled along the orchard floor in a standardized manner. Four individual swabs were pooled and a procedure designed for recovering *Salmonella* from poultry houses was used to enrich the samples. Three of six (50%) pooled swab samples collected in 2001 and 53 of 228 (23%) samples collected between 2002 and 2006 were positive for *Salmonella*; every isolate was identified as *Salmonella* Enteritidis PT 30. A total of 30 *Salmonella* Enteritidis PT 30 orchard isolates from 2001 (3), 2002 (12), 2003 (10), 2005 (2), and 2006 (3) were analyzed in the present study (S2 Table in S1 File). One orchard isolate from 2002 and all orchard isolates from 2004 (25) were not available and thus not included.

Using the pathogen detection tool associated with the NCBI database (https://www.ncbi.nlm.nih.gov/pathogens), the sequence read archive (SRA) data were downloaded for 12 *Salmonella* Enteritidis PT 30 clinical isolates from the 2001 almond outbreak and for four clinical isolates from the 2006 almond outbreak (S3 Table in S1 File).

### Whole genome sequencing

Isolates were retrieved from frozen glycerol stock and plated on tryptic soy agar (TSA). Following overnight incubation at 37°C, one colony was inoculated into 2 ml of tryptic soy broth (TSB) and incubated for 24 h at 37°C, with shaking at 120 rpm, before being pelleted by centrifugation at 14,000 × g for 2 min. Genomic DNA, for the isolates in S2 Table (S1 File), was extracted with the QIAamp DNA minikit (Qiagen, Valencia, CA) following the manufacturer’s directions. The 150-bp paired-end libraries were constructed for each purified DNA with the Illumina Nextera DNA flex library kit following the manufacturer’s directions (Illumina Inc., San Diego, CA). Pooled samples were sequenced on an Illumina 4000 HiSeq system by the DNA Technologies and Expression Analysis Core at the UC Davis Genome Center. DNA, for all the survey isolates, was sequenced as described by Emond-Rheault et al. in 2020 [16]. All sequence data obtained in this study were deposited to the NCBI pathogen database under the BioProject accession number PRJNA941918 (Tables 1 and S2 in S1 File) and PRJNA951760 (S2 Table in S1 File).

### Quality control and genome assembly

Raw read quality was assessed with FastQC (v0.11.8) (https://www.bioinformatics.babraham.ac.uk/projects/fastqc/). Illumina adapter sequences and low-quality sequences were trimmed using Trimmomatic version 0.36 [17]. Reads were assembled de novo with SPAdes version v3.13.0 Genome Assembler [18]. Draft *Salmonella* genome assemblies were serotyped with SeqSero [19] and by aligning with BLASTn against the nonredundant nucleotide sequence database.

### Core genome SNP typing

De novo genomes were used to build core genome single nucleotide trees using the Parsnp aligner v1.2 with default parameters and the requirement for all genomes to be included in the analysis [20]. The 171 *Salmonella* genomes from the survey were mapped to the complete reference genome *Salmonella* Typhimurium str. LT2 (NCBI accession: AE006468), resulting in an alignment of 50% of the core genome. The whole-genome phylogeny was constructed with FastTree2 [21]. iTOL (http://itol.embl/de/) was used to visualize the tree and annotate it with the AMR genes [22].

### Genetic distance

Genetic distance between multiple isolates of the same serovar was evaluated as the number of SNP differences detected with the CFSAN SNP Pipeline [12]. The CFSAN SNP Pipeline v2.0.2 was installed on a local ubuntu platform with all the executable software dependencies. Prior to analyzing our data, we used the data set provided for testing the reproducibility of the software to confirm that our installation of the CFSAN SNP Pipeline was correct. Reference-based alignments were created for a set of samples and used to generate the SNP matrix. Because the software was developed for closely related genome sequences, where available, complete assembled reference genomes were downloaded from NCBI (20; S4 Table in S1 File). The phylogenies were inferred with MEGA7 [23] using the neighbor-joining method [24] based on the obtained SNP matrix formatted as a FASTA file generated by the CFSAN SNP pipeline v2.0.2.

### Virulence and antibiotic resistance (AMR) gene prediction

The presence of resistance genes, as well as point mutations, were determined using ResFinder 4.1 (Center for Genomic Epidemiology, https://cge.food.dtu.dk/services/ResFinder) with a setting threshold of 90% and minimum length of 60% [25–27]. The assembled draft genomes for the survey isolates (171) were used as an input to identify *Salmonella* AMR genes associated with resistance to aminoglycoside, β-lactam, chloramphenicol, colistin, fluoroquinolone, fosfomycin, glycopeptide, macrolide, sulfonamide, tetracycline, and trimethoprim antibiotics. The VF analyzer pipeline was used to screen the assembled draft genomes against the Virulence Factor Database (VFDB) for potential virulence factors [28].

### Plasmid detection and reconstruction

Plasmids from genome assemblies were typed and reconstructed using MOB-suite v3.1.0 with the default parameters [29, 30]. To determine the AMR and virulence genes that were plasmid-borne, the reconstructed plasmids were screened against the CARD (https://card.mcmaster.ca) and VFDB [28] databases, respectively, using Abricate v0.5 (https://github.com/tseemann/abricate) with the same parameters as described above.

## Results

### Phylogenetic analysis of *Salmonella* isolates retrieved from raw almonds

**Fig 1:**
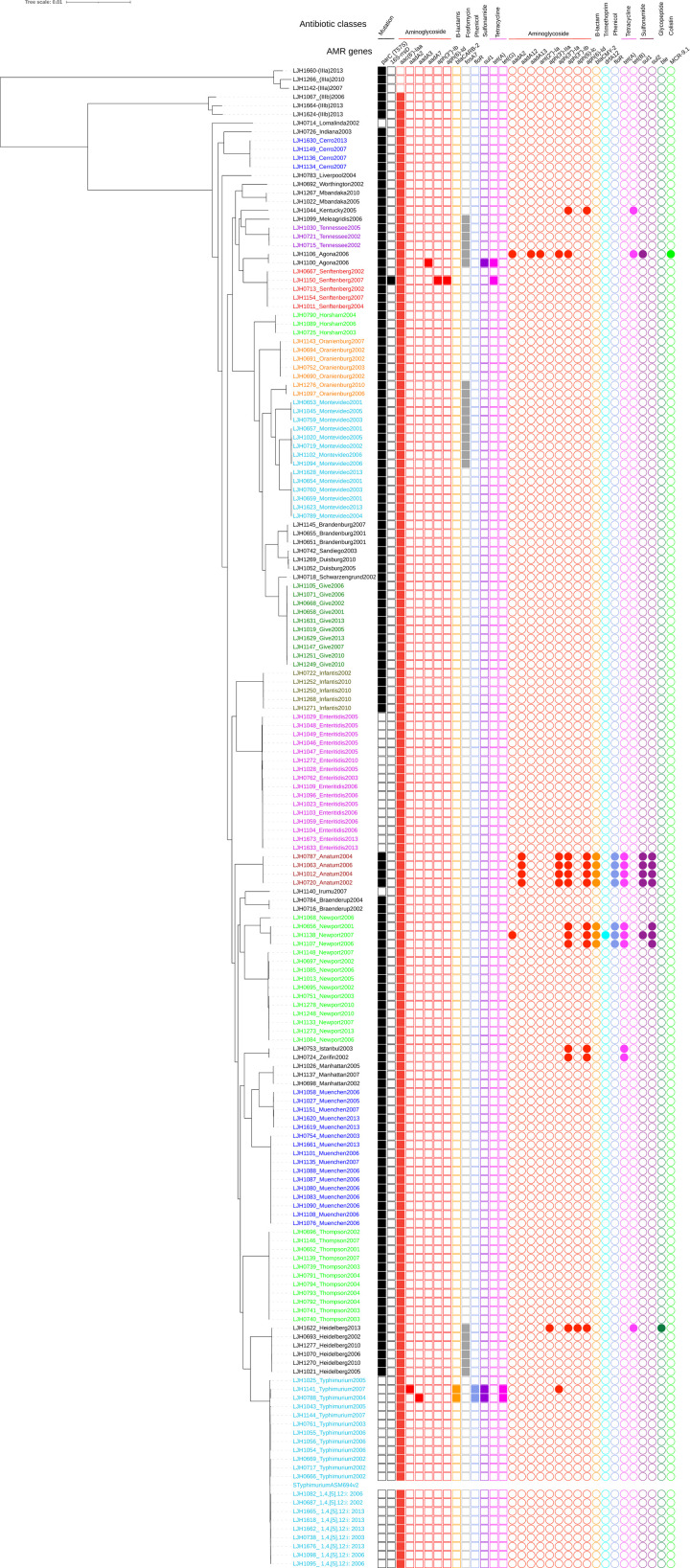
Maximum likelihood tree based on SNPs identified by aligning 171 *de novo* assemblies to the reference chromosome of *Salmonella* Typhimurium LT2 ASM694v2 with Parsnp and genotypic antimicrobial resistance (AMR). The scale bar is in number of SNPs. The colors in the phylogenetic tree represent different serogroups; squares represent a chromosomal location and circles represent a plasmid location for AMR genes. AMR genes are color-coded by antibiotic classes.

Genomes were initially compared with Parsnp because the CFSAN SNP Pipeline is not recommended for relatively distant bacteria (greater than a few hundred SNP differences). A maximum-likelihood phylogeny tree was constructed with Parsnp based on alignment of the 171 *Salmonella* assembled genomes [20]. All the isolates belonged to the species *enterica*, with most (165) belonging to the subspecies *enterica* (Fig 1 and Table 1). Three isolates were classified as subspecies *diarizonae* and three as subspecies *arizonae*. The phylogenetic tree clustered the isolates by serovar (Fig 1). The serovars identified by classical serotyping matched the serovars predicted by WGS for 92% of the isolates (Table 1). However, 14 isolates clustered with serotypes that differed from those to which they were initially assigned by traditional serotyping (Table 1). To eliminate the possibility of manipulation errors, these isolates were resequenced and their serotype was confirmed with SeqSero and with BLASTn against the nonredundant nucleotide sequence database. Results were consistent with the initial WGS serovar prediction.

Based on the core genome SNP typing, a total of 32 unique *Salmonella* serovars were identified. Of these, 22 *Salmonella* serovars were isolated two or more times between 2001 and 2013: Enteritidis (*n* = 16), Muenchen (*n* = 16), Newport (*n* = 15), Montevideo (*n* = 14), Typhimurium (*n* = 12), Thompson (*n* = 11), Give (*n* = 10), 1,4,[5],12:i- (*n* = 9), Oranienburg (*n* = 7), Heidelberg (*n* = 6), Infantis (*n* = 5), Senftenberg (*n* = 5), Anatum (*n* = 4), Cerro (*n* = 4), Brandenburg (*n* = 3), Duisburg (*n* = 3), Horsham (*n* = 3), Manhattan (*n* = 3), Tennessee (*n* = 3), Agona (*n* = 2), Braenderup (*n* = 2), and Mbandaka (*n* = 2) (Fig 1 and Table 1). Three isolates initially identified as *Salmonella* Othmarschen (LJH0752, LJH1143, and LJH1276-1) clustered with *Salmonella* Oranienburg. Both serotypes have a similar antigenic formula, 6,7,14:m,t:-, which makes them difficult to distinguish by serological methods [31]. Serovar 1,4,[5],12:i:- is a monophasic variant of *Salmonella* Typhimurium, and three of 14 isolates initially identified as Typhimurium (LJH0738, LJH1662-1, LJH1676) clustered with 1,4,[5],12:i:-. One *Salmonella* initially identified as 1,4,[5],12:i:- (LJH1144) clustered with Typhimurium.

Among the five isolates that were untypeable by classical serotyping, two clustered with serovar Muenchen (LJH1151, LJH1620-1), one with Enteritidis (LJH1029), and one with Manhattan (LJH1026). A single untypeable isolate (LJH1624-1) clustered with *Salmonella diarizonae* (LJH1664) and was identified as *Salmonella diarizonae* with BLASTn and SeqSero [19]. Fifteen unique serotypes, predicted with BLASTn and SeqSero, matched the serological serotyping. It was difficult to predict some serovars due to the limited number of representative genomes in the SeqSero database. *Salmonella* Zerifin LJH0724 was identified as *Salmonella* Istanbul with SeqSero and clustered with the Istanbul isolates in the Parsnp phylogeny tree (Fig 1). Conflicting serotyping results between traditional methods and WGS have been reported in previous studies [32–35]. Serotyping based on WGS has been increasingly used by public health laboratories and federal agencies to replace the current standard of phenotypic serotyping [10, 32, 34, 36]. In the present study, serotype was assigned based on the WGS analysis when the serovar assignment was identical among the phylogenetic relationship, SeqSero, and BLASTn analyses. Except for a single strain of *Salmonella* Zerifin, all isolates (170 of 171) were assigned a serotype based on the WGS analysis.

### Genetic distance within each serovar

**Fig 2:**
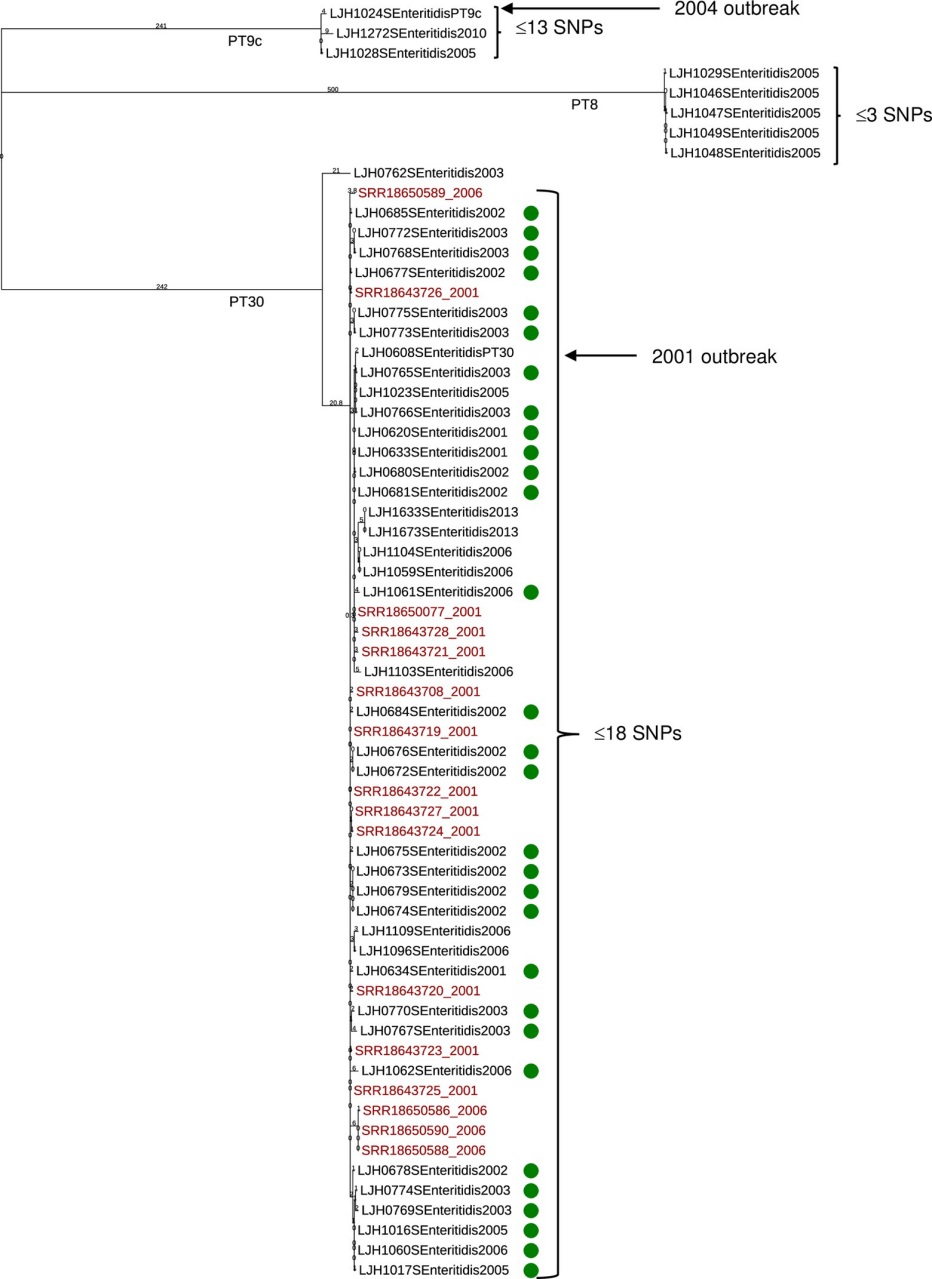
Phylogenetic tree of *Salmonella* Enteritidis generated with the CFSAN SNP Pipeline. The branch lengths represent the SNP distances among the isolates. Almond survey isolates (black text) and outbreak-associated orchard isolates (green dot) were compared to clinical isolates (red text) from the 2001 and 2006 outbreaks (retrieved from the NCBI database). Almond isolates of *Salmonella* Enteritidis PT 9c (LJH1024) and *Salmonella* Enteritidis PT 30 (LJH0608) from the 2004 and 2001 outbreaks, respectively, were included for comparison.

The CFSAN SNP Pipeline was used to evaluate the genetic distance among multiple isolates within each serotype. *Salmonella* Enteritidis isolates clustered in three groups by phage type: PT 8, PT 9c, and PT 30 (Fig 2). All five *Salmonella* Enteritidis PT 8 isolates were retrieved in 2005 from separate almond lots and had less than three SNP differences, classifying them as clonal isolates. *Salmonella* Enteritidis PT 9c isolated in 2005 (LJH1028) and 2010 (LJH1272), differed by 5 and 13 SNPs, respectively, from a 2004 *Salmonella* Enteritidis PT 9c outbreak isolate (LJH1024; Fig 2).

The genomes of *Salmonella* Enteritidis PT 30 recovered from survey almonds (eight isolates (Table 1): LJH0762 [2003], LJH1023 [2005], LJH1104 [2006], LJH1059 [2006], LJH1096 [2006], LJH1109 [2006], LJH1633 [2013], LJH1673 [2013]), the 2001 outbreak-associated orchard (30 isolates; S2 Table in S1 File), and a 2001 outbreak-associated almond isolate (LJH0608) were compared to *Salmonella* Enteritidis PT 30 genomes of clinical isolates from almond outbreaks in 2001 (12 isolates) and 2006 (four isolates) (S3 Table in S1 File).

*Salmonella* Enteritidis PT 30 isolates formed two clusters (Fig 2). One consisted of a single survey isolate (LJH0762), recovered in 2003, that differed from LJH0608 by 48 SNPs (Fig 2 and S5 Table in S1 File). All other survey and clinical isolates (*n* = 38) clustered in a single group with LJH0608 that differed from each other by ≤18 SNPs (Fig 2) indicating that the isolates are from a common origin. Almond isolates from 2001 to 2013 had 2 to 13 SNP differences compared with the 2001 outbreak-associated almond isolate *Salmonella* Enteritidis PT 30 LJH0608 (Fig 2 and S5 Table in S1 File). Although this isolate was recovered from recalled almonds in 2001, the almonds were harvested in the fall of 2000 [1], a span of 14 years (2000–2013). The orchard isolates from 2001 to 2006 differed by 0 to 12 SNPs within their genomes and by 3 to 13 SNPs with the clinical genomes. The SNP differences ranged from zero to eight within the 12 clinical isolates from the 2001 outbreak and from one to 13 within the four clinical isolates from the 2006 outbreak. Among the clinical isolates from 2001 and 2006, the SNP differences ranged from four to 13, indicating that the isolates are from a common origin.

Almond, orchard, and clinical isolates of *Salmonella* Enteritidis PT 30 isolated from 2001 through 2013 are closely related strains. The persistence of *Salmonella* Enteritidis PT 30 in an almond orchard over 6 years was reported previously [2]. The SNP analysis confirmed the PFGE results obtained for these isolates. Almonds from the 2001 outbreak-associated orchards were purposefully excluded from the raw almond survey. Because survey samples were coded and the sources unknown, it is possible that samples harvested from outbreak-associated orchards were inadvertently included. It is also possible that survey almonds were cross contaminated with almonds harvested from outbreak-associated orchards via harvest equipment or at a common almond huller-sheller, or that *Salmonella* Enteritidis PT 30 was spread over a broader geographic region than recognized as associated with the 2001 outbreak.

**Fig 3:**
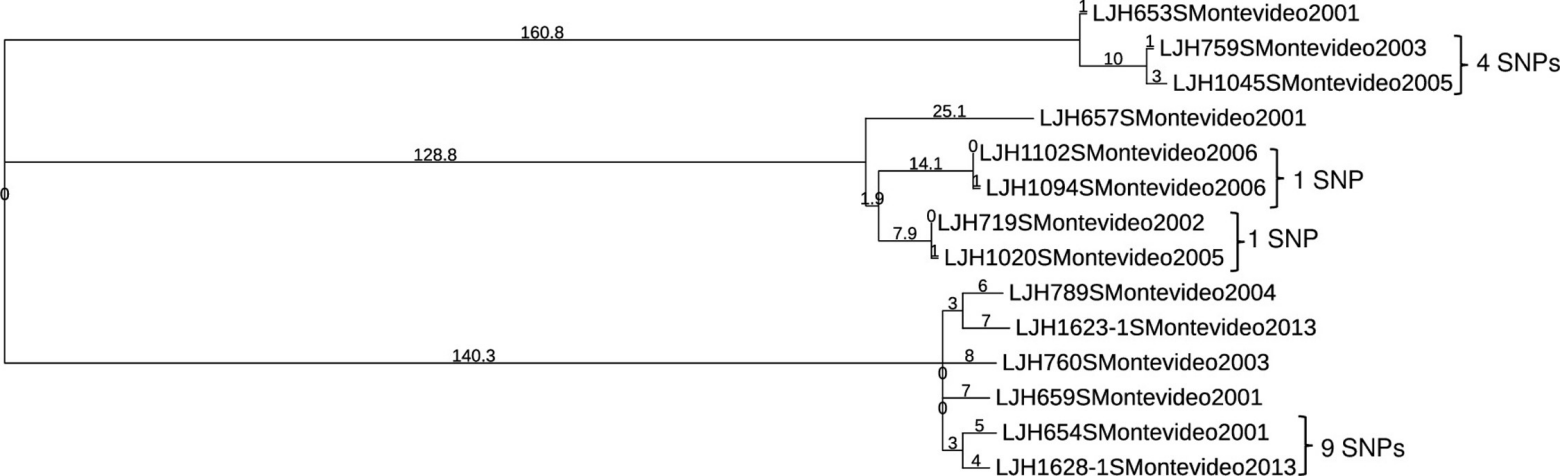
Phylogenetic tree of *Salmonella* Montevideo generated with the CFSAN SNP Pipeline. The branch lengths represent the SNP distances among the isolates.

**Fig 4:**
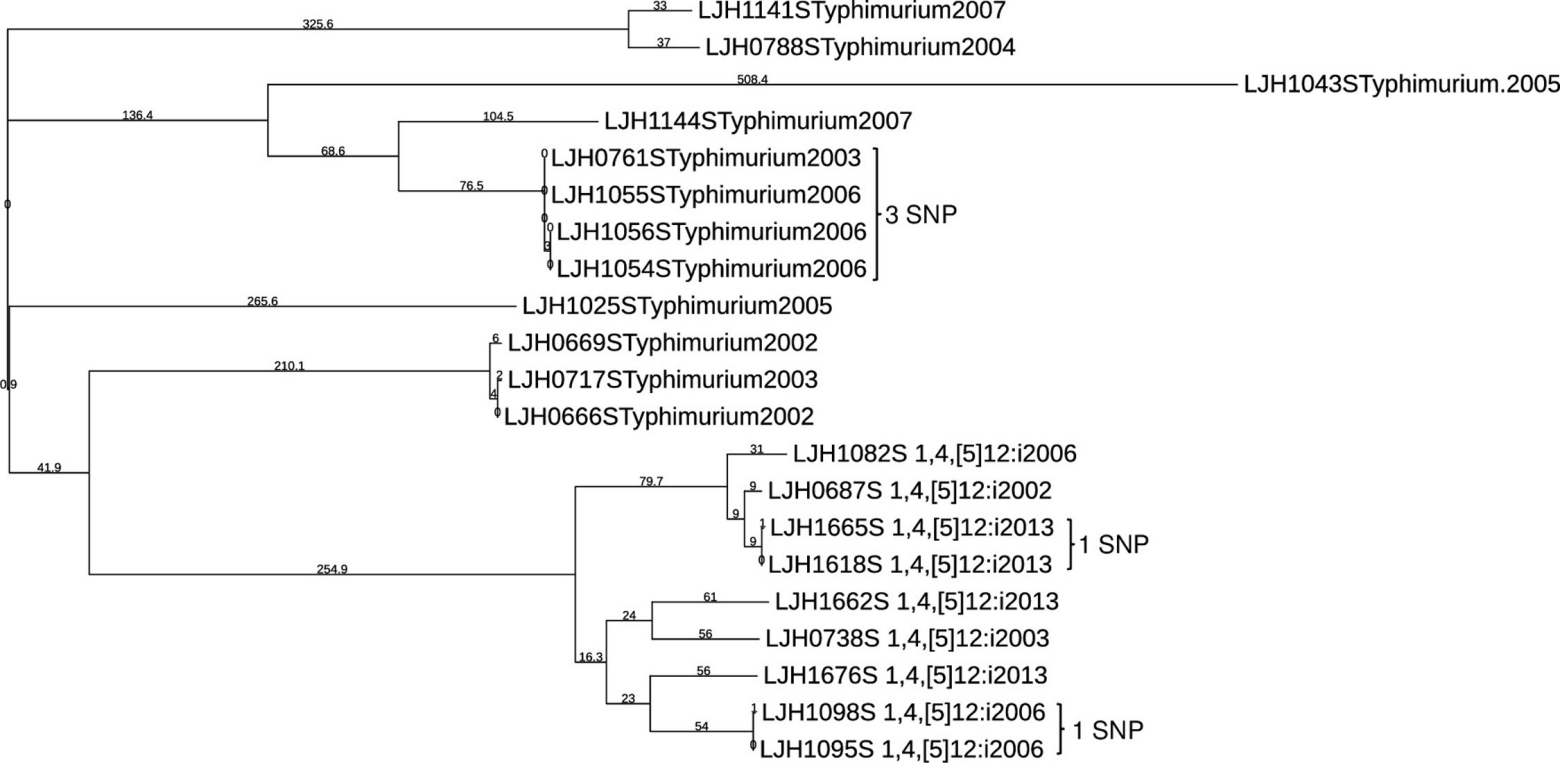
Phylogenetic tree of *Salmonella* Typhimurium and 1,4,[5],12:i:-. The branch lengths are representative of the SNP distances among the isolates.

*Salmonella* Enteritidis was not the only serovar for which clonal isolates were recovered from almonds in different years. *Salmonella* Montevideo survey isolates clustered into three groups, with more than 100 SNPs between them (Fig 3). Within each of these clusters there were isolates separated by one or more years (including isolates from 2001 and 2013) that differed by ≤9 SNPs, indicating that they share a common ancestor. *Salmonella* Newport (S6 Table in S1 File) and *Salmonella* Muenchen (S7 Table in S1 File) each clustered into two groups separated by more than 100 SNPs. A small number of SNP differences (<3) between *Salmonella* Newport genomes (S6 Table in S1 File) were identified in isolates retrieved in 2003 (LJH0751), 2006 (LJH1084), 2007 (LJH1133), 2010 (LJH1248, LJH1273 and LJH1278). Nine isolates of *Salmonella* Muenchen were retrieved in 2006; eight of these isolates from six different almond lots had nearly identical genomes, with ≤3 SNP differences (S7 Table in S1 File), and differed from single isolates in 2007 (LJH1135) and 2013 (LJH1661) by ≤8 SNPs. Closely related genomes for single isolates of *Salmonella* Anatum (S8 Table in S1 File) and *Salmonella* Thompson (S9 Table in S1 File) were identified 2 and 1 years apart, respectively. Several *Salmonella* Oranienburg (S10 Table in S1 File) and *Salmonella* Typhimurium (Fig 4) were identified 5 and 3 years apart, respectively. Closely related genomes (≤13 SNPs) were identified for isolates retrieved from separate almond lots during the same year for *Salmonella* serovar Cerro (S11 Table in S1 File), Give (S12 Table in S1 File), Infantis (S13 Table in S1 File), Heidelberg (S14 Table in S1 File) and Tennessee (S15 Table in S1 File). For *Salmonella* serovar Brandenburg (S16 Table in S1 File), Braenderup, Manhattan (S17 Table in S1 File), Mbandaka, and Senftenberg (S18 Table in S1 File), isolates were separated by more than 13 SNPs.

Multiple SNP-based approaches have been developed to analyze the large number of short reads produced by various sequencing platforms [37, 38]. The CFSAN Pipeline was selected because it is less sensitive to coverage changes [37] and has good discriminative power. Its high resolution, however, strongly depends on an appropriate reference genome. In addition to outbreak source attribution, this tool can reveal similarity among isolates and their persistence in food facilities or in the production environment [14, 39].

Differences of more than 100 SNPs were sometimes observed among isolates within each serovar. Survey isolates that were genetically similar (≤13 SNPs) were recovered in multiple years, up to 13 years for *Salmonella* Enteritidis PT 30 and *Salmonella* Montevideo and 10 years for *Salmonella* Newport. Because the survey samples were collected after hulling and shelling and prior to entering a processing facility or storage, the contamination source would have to be at production or harvest (orchard), during postharvest handling (transportation to huller, during storage, or during hulling and shelling), or transportation from huller to processor. Although the exact geographic locations of the almond survey samples were unknown, clonal strains of *Salmonella* Enteritidis PT 8 (five; 2005) and *Salmonella* Muenchen (eight; 2006) were isolated from different lots of almonds in the same year.

The diversity of serovars and unique strains within serovars was expected in a survey that likely reflects broad environmental contamination (e.g., almond orchard during production or harvest). Common harvest and postharvest practices may also lead to distribution of *Salmonella* in almonds from geographically diverse orchards that share the same equipment or facilities. At maturity, almonds are shaken to the ground where they dry for several days. They are then harvested by windrowing and sweeping off the orchard floor. The in-hull, inshell almonds are then transported to facilities where the hull and shell are removed. Kernels mix with hulls and shells before sorting, separation, and bulk transportation to processing facilities. Once kernels are delivered to almond processing facilities, commingling of almond lots may occur prior to or during storage. These practices may explain the clusters of *Salmonella* Enteritidis PT 8 and *Salmonella* Muenchen isolated from different lots in 2005 and 2006.

U.S. regulations were implemented in 2007 that require all California-grown almonds sold in North America (U.S., Canada, and Mexico) to be processed with a treatment capable of achieving a minimum 4-log reduction in *Salmonella* [40]. While there have been outbreaks associated with almond-containing products such as blended nut butters, none have been associated with contaminated almonds since 2006, likely due to effective implementation of these regulations [7, 15].

The persistence of *Salmonella* has been described for other pre- and postharvest scenarios [11, 41, 42]. A narrow range of *Salmonella* serovars has been associated with California pistachio outbreaks, outbreak investigations, and industry and retail surveys [41, 43]. Pistachio-associated isolates of *Salmonella* Senftenberg and *Salmonella* Montevideo recovered over multiple years (2009 to 2017) and from multiple facilities differed (within each serovar) by 0 to 31 SNPs, and the authors suggested that the organisms may have established residence in the primary production environment or orchards [41].

### Plasmid prediction and characterization

**Fig 5:**
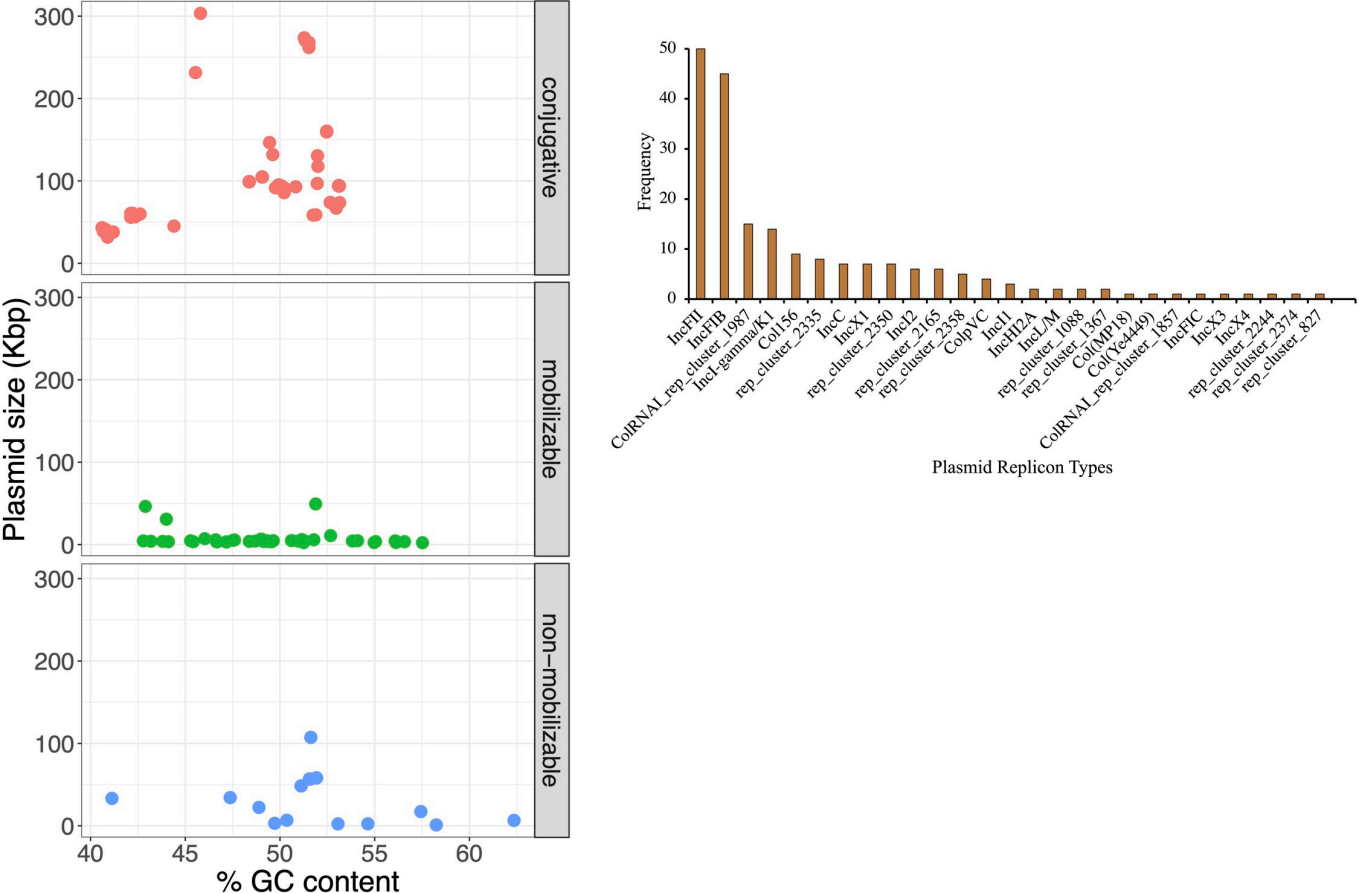
General features of plasmids identified in *Salmonella* survey isolates included in this study. (A) Putative plasmids predicted were categorized as mobilizable, conjugative, or non-mobilizable based on the presence or absence of relaxase, mate-pair formation marker genes and/or *oriT* sequence. Each colored circle represents a plasmid-carrying *Salmonella* isolate. (B) Frequency of plasmid replication types.

Plasmids contribute significantly to the emergence and spread of genes encoding AMR, virulence, and other metabolic functions in multiple scales across *Salmonella* serotypes [16, 44]. Plasmid carriage in the *Salmonella* strains under study were assessed by screening and reconstructing the plasmid sequences from assembled genomes using the clustered plasmid reference database-based pipeline [29, 30]. A total of 106 of the 171 *Salmonella* isolates (62%) carried one to seven plasmids (total plasmids 161; Table 1) with sizes of 1,030 bp to 303,322 bp (Fig 5A and S19 Table in S1 File). Using the presence of relaxase and mate-pair formation marker genes and/or *oriT* sequence, most of these plasmids were predicted to be either mobilizable (*n* = 51/161; 32%) or conjugative (*n* = 83/161; 52%) (Fig 5A). In total, 61 plasmid clusters with 27 different plasmid families were identified, with IncFII and IncFIB being the most predominant types in the collection (Fig 5B).

**Fig 6:**
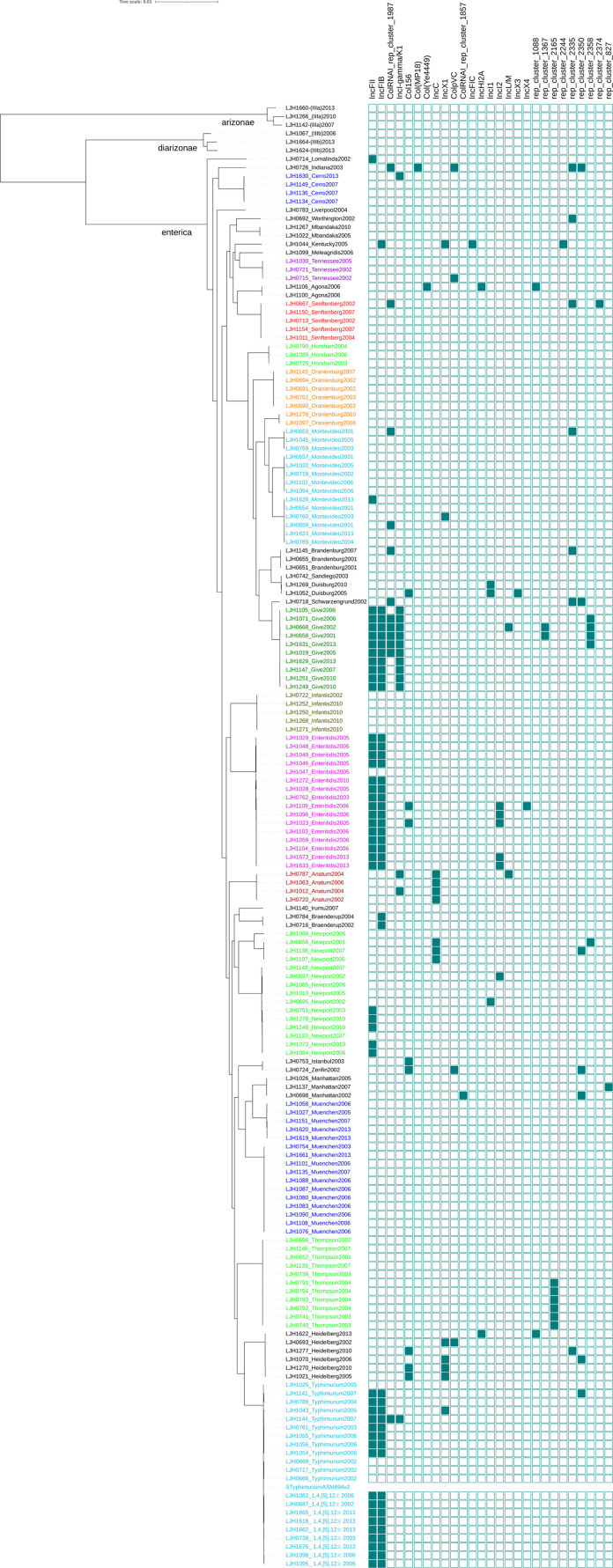
Heatmap of the distribution of plasmid replication types detected in *Salmonella* isolates.

Thirty eight *Salmonella* strains distributed across six serovars (Duisburg, Enteritidis, Lomalinda, Muenchen, Typhimurium, and 1,4,[5],12:i:-) carried plasmids that contained two to seven virulence genes, but no AMR genes (S20 Table in S1 File). The IncFIB-IncFII plasmid family combination was common among these strains (Fig 6), predominantly in *Salmonella* Enteritidis (*n* = 15), Typhimurium (*n* = 8), and 1,4,[5],12:i:- (*n* = 9). Comparative analysis of the plasmid sequences with known plasmids using mash [45] and BLASTn revealed that these plasmids are similar (nucleotide sequence homology = 100%) to plasmid pCFSAN076214_2 (accession number CP033342.1) described previously in *Salmonella* Enteritidis strain ATCC BAA-1045 isolated from raw almonds (also LJH608 in this study) [46] and to plasmid p11-0972.1 (accession number CP039855.1) reported in *S*. *enterica* serovar 1,4,[5],12:i:- recovered from a human stool sample [47]. IncF plasmids are among the most common plasmids found in *Salmonella* and are reported to carry multiple antibiotic resistance and/or virulence genes, suggesting their role in the dissemination of these genes across *Salmonella* serotypes and Enterobacteriaceae by extension [48, 49].

Thirteen *Salmonella* strains belonging to seven serotypes (Agona, Anatum, Heidelberg, Istanbul, Newport, Typhimurium, and Zerifin) carried plasmids with at least one AMR gene but no virulence genes. The most common among these was within the IncC plasmid family and was associated with six to 10 AMR genes (S20 Table in S1 File). The IncC plasmid identified in the present study was predicted to be conjugative, predominant in *Salmonella* Anatum and *Salmonella* Newport (Fig 6) and was almost indistinguishable (99.99% by BLASTn) from *Salmonella* Anatum plasmid pSAN1-1736 (accession number: CP014658.1) [50]. IncC plasmids are known to be widely distributed across *Salmonella* serotypes from diverse sources and often carry multiple AMR genes [49].

A unique *Salmonella* Kentucky strain LJH1044 carried a large conjugative plasmid (146 Kb) with IncFIB-IncFIC-rep_cluster_2244 plasmid replicons and contained multiple virulence and AMR genes (Fig 6 and S20 Table in S1 File). This plasmid carried three AMR genes encoding resistance to aminoglycoside and tetracycline, a complete *iroBCDEN* operon that encodes salmochelin siderophore [51], and an *iucABCD-iutA* operon that has been described to be associated with aerobactin synthesis essential for virulence and stress response in *Salmonella* [52] (S20 Table in S1 File). Comparative analysis showed that this plasmid had 99.99% nucleotide sequence similarity to plasmid pCVM29188_146 (accession number CP001122.1) reported previously in several *Salmonella* Kentucky strains from poultry in the United States [53, 54].

### Antimicrobial resistance profile

Among the isolates retrieved from the almond survey, a total of 24 AMR genes were identified with the ResFinder and CARD database, which classified into nine different antimicrobial protein groups: aminoglycosides, β-lactams, colistin, fosfomycin, glycopeptide, phenicol, sulfonamide, tetracycline, and trimethoprim. One gene, *aac(6’)-Iaa*, which confers resistance to aminoglycosides, was detected in the chromosome of all *Salmonella* subspecies *diarizonae* (3) and *enterica* (165) but not in *arizonae* (3) (Fig 1). The frequency of *Salmonella enterica* subspecies *enterica* isolates that carried an aminoglycoside acetyltransferase *aac(6’)-Iaa* has been reported to be high (>95%) in multiple WGS analysis [55–57]. However, in a surveillance study of non-typhoidal *Salmonella enterica*, 11 isolates out of 3,491 (0.3%) showed phenotypic resistance to an aminoglycoside antimicrobial [55].

The other 23 AMR genes were detected in 35 isolates, with *fosA7* being the most common (Fig 1). The gene *fosA7*, which confers resistance to fosfomycin, a broad-spectrum cell wall synthesis inhibitor, was first identified in the chromosome of *Salmonella* Heidelberg isolated from broiler chickens [58]. In the present study, all *Salmonella* Heidelberg isolates (*n* = 6), and some isolates of *Salmonella* serovars Agona (*n* = 2), Meleagris (*n* = 1), Montevideo (*n* = 8), Oranienburg (*n* = 2), and Tennessee (*n* = 3), carried the chromosomal *fosA*7 gene. Ten aminoglycoside resistance genes were detected in 16 isolates: *aac(6’)-Iaa*, *aadA2*, *aadA3*, *aadA7*, *aadA12*, *aadA13*, *ant(2”)-Ia*, *aph(3")-IIa*, *aph(3’’)-Ib*, *aph(6)-lc*, and *aph(6)-*Id. The *bla*_*CMY-2*_ and *bla*_*CARB-2*_ genes, which confer resistance to β-lactams, and the *floR* gene, which confers resistance to phenicol, were found in nine isolates. Three tetracycline efflux resistance genes were identified in 16 isolates: *tetA*, *tetB*, and *tetG*. Sulfisoxazole resistance, encoded by *sul1* or *sul2*, was detected in 11 isolates. The dihydrofolate reductase resistance gene, *dfrA12*, which confers resistance to trimethoprim, was detected in one *Salmonella* Newport isolate. The plasmid-mediated colistin resistance and phosphoethanolamine transferase *mcr*-9.1 gene was detected in one *Salmonella* Agona isolate. Resistance to bleomycin, encoded by the bleomycin-binding protein gene (*ble*), was predicted for one *Salmonella* Heidelberg isolate. All the isolates, except *Salmonella* serovars Enteritidis, Irumu, Lomalinda, Typhimurium, and 1,4,[5],12:i-, contained a missense mutation in *parC* associated with resistance to quinolone. Multiple mutations in the quinolone resistance determining region are usually required to confer resistance to ciprofloxacin, but one mutation confers resistance to nalidixic acid [59]. One *Salmonella* Senftenberg isolate had a point mutation in the 16S rRNA that is thought to confer resistance to spectinomycin.

Most of the predicted AMR genes were identified in a small number (16 out of 171; 9%) of survey isolates and were plasmid encoded in 11 of 16 cases (Fig 1). Multidrug-resistant isolates (putative resistance to at least one antibiotic in three or more drug classes; https://www.cdc.gov/narms/resources/glossary.html) were identified among *Salmonella* serovars Agona (LJH1100 and LJH1106), Anatum (LJH0720, LJH0787, LJH1012, and LJH1063), Heidelberg (LJH1622), Newport (LJH0656, LJH1138, and LJH1107), and Typhimurium (LJH0788 and LJH1141) (Fig 1). Antibiotic resistance profiles by the calibrated dichotomous sensitivity method were determined for *Salmonella* isolated from 2001 through 2005 but not for isolates from 2006, 2007, 2010, and 2013. Ten of the *Salmonella* survey isolates from 2001 to 2005 were resistant to three or more antibiotics [5]. Resistance genotype and phenotype correlated highly for five of these isolates: *Salmonella* Anatum (*n* = 3), *Salmonella* Istanbul (*n* = 1), and *Salmonella* Typhimurium var. Copenhagen (*n* = 1).

### Analysis of virulence factors

**Fig 7:**
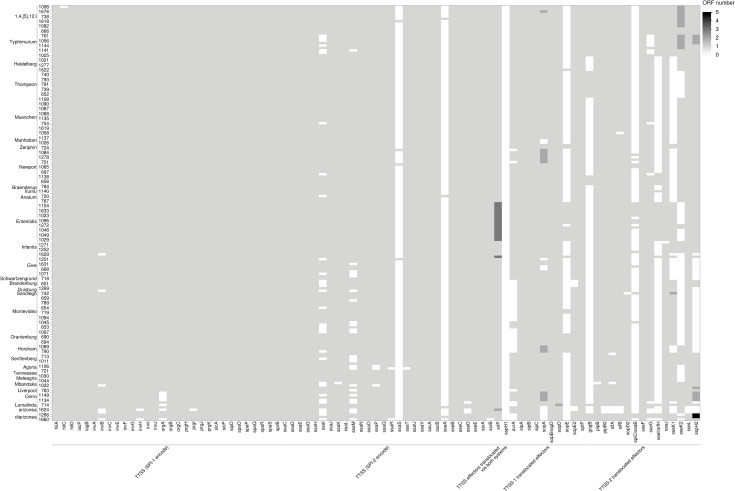
Heatmap of the distribution of virulence genes encoding type III secretion system (T3SS) across 171 genomes. The shades of gray represent the number of open reading frames (ORF) that are detected in each putative virulence gene.

**Fig 8:**
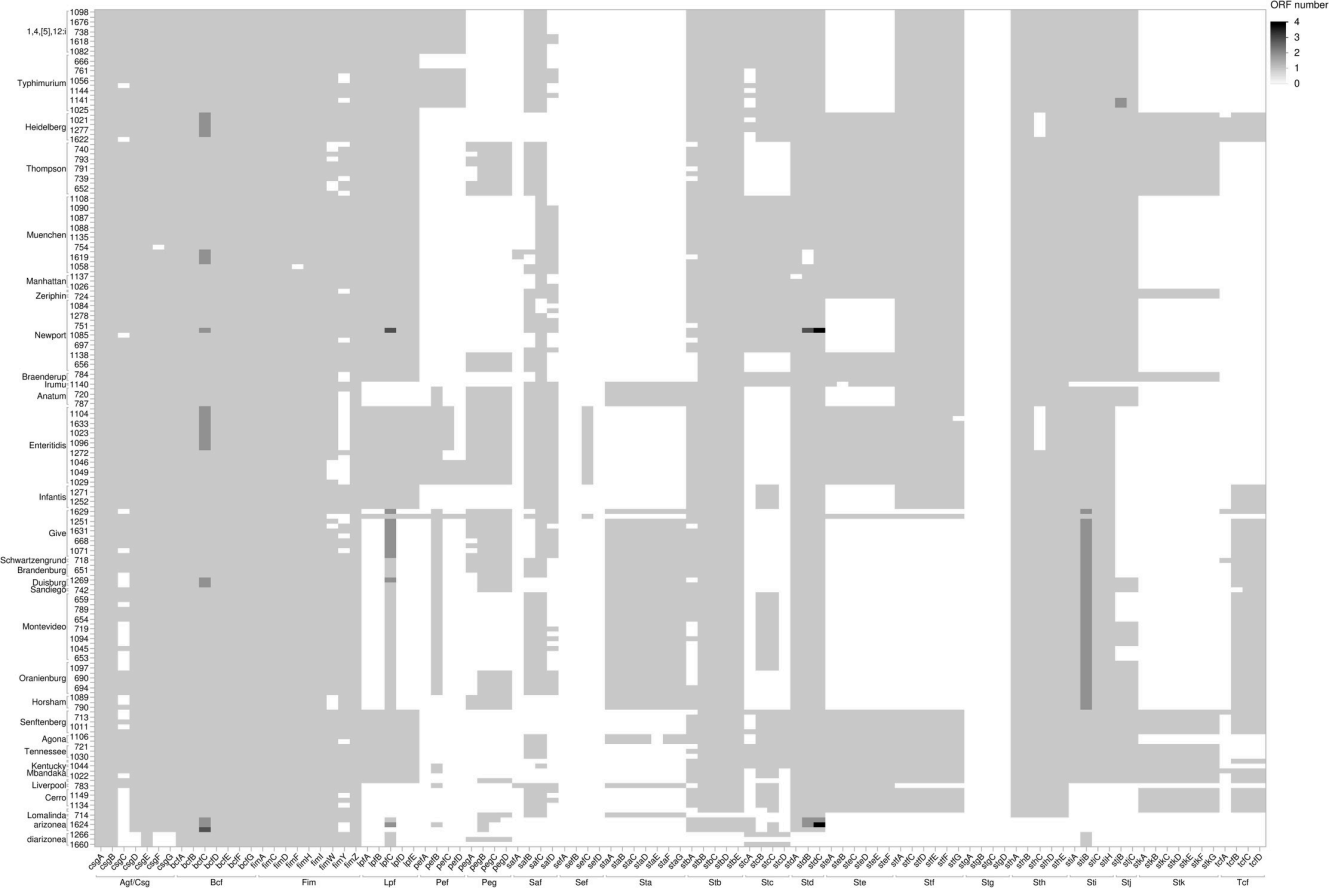
Heatmap of the distribution of genes encoding the fimbrial operon across 171 genomes. The shades of gray represent the number of open reading frames (ORF) that are detected in each putative virulence gene.

**Fig 9:**
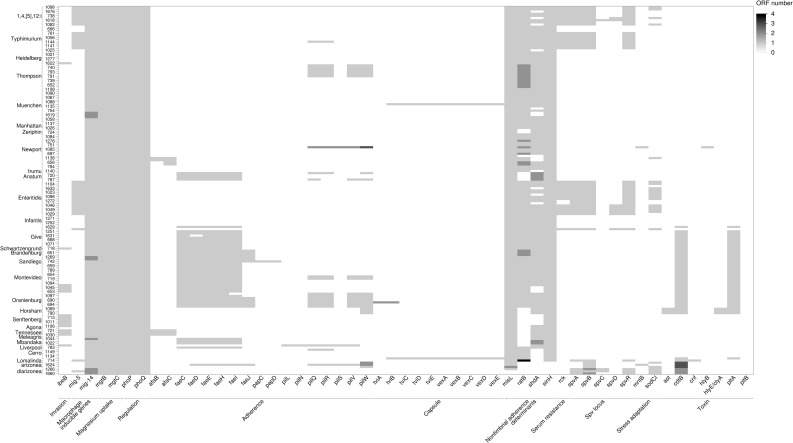
Heatmap of the distribution of other virulence genes across 171 genomes. The shades of gray represent the number of open reading frames (ORF) that are detected by in each putative virulence gene.

*Salmonella* serovars infect a wide range of hosts with different degrees of disease severity, with Enteritidis, Newport, Typhimurium, Javiana, and 1,4,[5],12:i- being significantly more likely to cause illness in humans in the United States [60]. Differences in virulence factors contribute to the severity and outcome of salmonellosis and can be specific to serovars [60]. The Virulence Factor Database (VFDB) was used to detect a total of 303 virulence genes among the 171 *Salmonella* assembled genomes (Figs 7–9). Genes were classified under major virulence factors, including the secretion system, fimbrial and non fimbrial adherence, macrophage inducible genes, magnesium uptake, serum resistance, stress proteins, toxins, immune invasion, and two component regulatory systems. The *Salmonella* pathogenicity island 1 (SPI-1) and 2 (SPI-2), responsible for the type III secretion system, are ubiquitous in *S*. *enterica* subsp. *enterica* [60] and were common to all 171 survey isolates (Fig 7).

Among the fimbrial adherence factors, the genes encoding the curli fimbriae, *csgA*, *csgB*, and *csgE*, the *bcfABCDEFG* operon, and the genes that encode type 1 fimbriae, *fimA*, *fimB*, *fimC*, *fimD*, *fimH*, *fimI*, *fimW*, and *fimZ*, were also present in all the isolates (Fig 8). The two- component regulatory system *phoP*-*PhoQ* genes and the magnesium uptake genes, *mgtB*-*mgtC*, (part of SPI-3) were present in all the isolates (Fig 9). All the isolates had the microphage inducible gene, *mig-14*, but *mig*-*5* was mainly detected in isolates of *Salmonella* serovars Enteritidis, Typhimurium, and 1,4,[5]12:I:- (Fig 9).

The typhoid toxin genes, *cdtB* and *pltA*, originally identified in serotype Typhi, were found in all *Salmonella* serovar Brandenburg, Duisburg, Horsham, Montevideo, Sandiego, and Schwartzengrund isolates and in nine of 10 *Salmonella* Oranienburg isolates (Fig 9). However, *pltB*, required for forming holotoxin, was not present in any of these isolates. In *Salmonella* Horsham isolates, two genes were identified as homologs of the enterotoxin hemolysin genes of *Escherichia coli* (*hylE*/*clyA*) (Fig 9).

A virulence plasmid that harbored a combination of *pef*, *rck*, and *spv* virulence genes was identified in 23% of the isolates (S20 Table in S1 File). The assembly of the major Pef fimbriae depends on the *pefBACDorf5orf6* operon, which encodes PefA fimbriae subunit, PefC usher protein, and the pefD periplasmic chaperone [61]. Eight of 10 *Salmonella* Typhimurium and all nine *Salmonella* 1,4,[5]12:I:- isolates carried a plasmid encoding *pefA*, *pefB*, *pefC*, and *pefD* genes (Fig 8 and S20 Table in S1 File).

The *spv* genes play a role in suppression of the innate immune response and are often associated with invasive disease and increased virulence [62, 63]. The *spv* genes were detected in all *Salmonella* serovar Lomalinda, Enteritidis, and 1,4,[5]12:I:- isolates, in eight of 10 *Salmonella* Typhimurium isolates, and one of 10 *Salmonella* Give isolates (Fig 9 and S20 Table in S1 File). The *rck* gene, which provides protection against the complement-mediated immune response of the host [64], was found in one of 10 *Salmonella* Give isolates, in all *Salmonella* Enteritidis PT 30 and PT 8 isolates, in eight of 10 *Salmonella* Typhimurium isolates, and all *Salmonella* 1,4,[5]12:I;- isolates (Fig 9).

This study provides one of the first in-depth longitudinal characterizations of *Salmonella* strains isolated from a single product (almonds) or production environment (almond orchard), in a single geographical region (Central California). This isolate collection is important for understanding *Salmonella* populations in a significant food production region of the United States. Several clonal strains of *Salmonella* were isolated over multiple years, adding to a growing body of evidence that enteric pathogens may persist over long periods of time (years) in agricultural environments and in postharvest or food processing facilities (https://www.cdc.gov/ncezid/dfwed/outbreak-response/rep-strains.html) [41, 42]).

## Supporting information

S1 File (XLSX)

## References

[1] Isaacs S, Aramini J, Ciebin B, Farrar JA, Ahmed R, Middleton D, et al. An international outbreak of salmonellosis associated with raw almonds contaminated with a rare phage type of *Salmonella* Enteritidis. J Food Prot. 2005;68: 191–198. doi: 10.4315/0362-028X-68.1.191 15690826

[2] Uesugi AR, Danyluk MD, Mandrell RE, Harris LJ. Isolation of *Salmonella* Enteritidis phage type 30 from a single almond orchard over a 5-year period. J Food Prot. 2007;70: 1784–1789. doi: 10.4315/0362-028X-70.8.1784 17803132

[3] Ledet Muller L, Hjertqvist M, Payne L, Pettersson H, Olsson A, Plym-Forshell L, et al. Cluster of Salmonella Enteritidis in Sweden 2005–2006 –suspected source: almonds. Eurosurveillance. 2007;12: 9–10. doi: 10.2807/esm.12.06.00718-en 17991404

[4] CDC Centers for Disease Control and Prevention. Outbreak of Salmonella serotype Enteritidis infections associated with raw almonds—United States and Canada, 2003–2004. MMWR Morb Mortal Wkly Rep. 2004: 53: 484–487. Available: https://www.cdc.gov/mmwr/preview/mmwrhtml/mm5322a8.htm. Accessed 11 Nov 2019. 15190247

[5] Danyluk MD, Jones TM, Abd SJ, Schlitt-Dittrich F, Jacobs M, Harris LJ. Prevalence and amounts of *Salmonella* found on raw California almonds. J Food Prot. 2007;70: 820–827. doi: 10.4315/0362-028X-70.4.820 17477248

[6] Bansal A, Jones TM, Abd SJ, Danyluk MD, Harris LJ. Most-probable-number determination of *Salmonella* levels in naturally contaminated raw almonds using two sample preparation methods. J Food Prot. 2010;73: 1986–1992. doi: 10.4315/0362-028X-73.11.1986 21219709

[7] Santillana Farakos SM, Pouillot R, Johnson R, Spungen J, Son I, Anderson N, et al. A quantitative assessment of the risk of human salmonellosis arising from the consumption of almonds in the United States: The impact of preventive treatment levels. J Food Prot. 2017;80: 863–878. doi: 10.4315/0362-028X.JFP-16-403 28414255

[8] Parker CT, Huynh S, Quinones B, Harris LJ, Mandrell RE. Comparison of genotypes of *Salmonella enterica* serovar Enteritidis phage type 30 and 9c strains isolated during three outbreaks associated with raw almonds. Appl Environ Microbiol. 2010;76: 3723–3731. doi: 10.1128/AEM.03053-09 20363782 PMC2876433

[9] Brown E, Dessai U, Mcgarry S, Gerner-Smidt P. Use of whole-genome sequencing for food safety and public health in the United States. Foodborne Pathog Dis. 2019;16: 441–450. doi: 10.1089/fpd.2019.2662 31194586 PMC6653787

[10] Stevens EL, Carleton HA, Beal J, Tillman GE, Lindsey RL, Lauer AC, et al. Use of whole genome sequencing by the Federal Interagency Collaboration for Genomics for Food and Feed Safety in the United States. J Food Prot. 2022;85: 755–772. doi: 10.4315/JFP-21-437 35259246

[11] Pightling AW, Pettengill JB, Luo Y, Baugher JD, Rand H, Strain E. Interpreting whole-genome sequence analyses of foodborne bacteria for regulatory applications and outbreak investigations. Front Microbiol. 2018;9: 1482. doi: 10.3389/fmicb.2018.01482 30042741 PMC6048267

[12] Davis S, Pettengill JB, Luo Y, Payne J, Shpuntoff A, Rand H, et al. CFSAN SNP Pipeline: an automated method for constructing SNP matrices from next-generation sequence data. PeerJ Comput Sci. 2015;1: e20. doi: 10.7717/peerj-cs.20

[13] Katz LS, Griswold T, Williams-Newkirk AJ, Wagner D, Petkau A, Sieffert C, et al. A comparative analysis of the Lyve-SET phylogenomics pipeline for genomic epidemiology of foodborne pathogens. Front Microbiol. 2017;8: 375. doi: 10.3389/fmicb.2017.00375 28348549 PMC5346554

[14] Wang YU, Pettengill JB, Pightling A, Timme R, Allard M, Strain E, et al. Genetic diversity of *Salmonella* and *Listeria* isolates from food facilities. J Food Prot. 2018;81: 2082–2089. doi: 10.4315/0362-028X.JFP-18-093 30485763

[15] Lambertini E, Danyluk MD, Schaffner DW, Winter CK, Harris LJ. Risk of salmonellosis from consumption of almonds in the North American market. Food Res Int. 2012;45: 1166–1174. doi: 10.1016/j.foodres.2011.05.039

[16] Emond-Rheault JG, Hamel J, Jeukens J, Freschi L, Kukavica-Ibrulj I, Boyle B, et al. The *Salmonella enterica* plasmidome as a reservoir of antibiotic resistance. Microorganisms. 2020;8: 1016. doi: 10.3390/microorganisms8071016 32650601 PMC7409225

[17] Bolger AM, Lohse M, Usadel B. Trimmomatic: a flexible trimmer for Illumina sequence data. Bioinformatics. 2014;30: 2114–2120. doi: 10.1093/bioinformatics/btu170 24695404 PMC4103590

[18] Bankevich A, Nurk S, Antipov D, Gurevich AA, Dvorkin M, Kulikov AS, et al. SPAdes: A new genome assembly algorithm and its applications to single-cell sequencing. J Comput Biol. 2012;19: 455–477. doi: 10.1089/cmb.2012.0021 22506599 PMC3342519

[19] Zhang S, Yin Y, Jones MB, Zhang Z, Deatherage Kaiser BL, Dinsmore BA, et al. *Salmonella* serotype determination utilizing high-throughput genome sequencing data. J Clin Microbiol. 2015;53: 1685–92. doi: 10.1128/JCM.00323-15 25762776 PMC4400759

[20] Treangen TJ, Ondov BD, Koren S, Phillippy AM. The Harvest suite for rapid core-genome alignment and visualization of thousands of intraspecific microbial genomes. Genome Biol. 2014;15: 524. doi: 10.1186/s13059-014-0524-x 25410596 PMC4262987

[21] Price MN, Dehal PS, Arkin AP. FastTree 2 –Approximately maximum-likelihood trees for large alignments. PLoS One. 2010;5: e9490. doi: 10.1371/journal.pone.0009490 20224823 PMC2835736

[22] Letunic I, Bork P. Interactive Tree Of Life (iTOL) v4: recent updates and new developments. Nucleic Acids Res. 2019;47: W256–W259. doi: 10.1093/nar/gkz239 30931475 PMC6602468

[23] Kumar S, Stecher G, Tamura K. MEGA7: Molecular evolutionary genetics analysis version 7.0 for bigger datasets. Mol Biol Evol. 2016;33: 1870–1874. doi: 10.1093/molbev/msw054 27004904 PMC8210823

[24] Saitou N, Nei M. The neighbor-joining method: a new method for reconstructing phylogenetic trees. Mol Biol Evol. 1987;4: 406–425. doi: 10.1093/oxfordjournals.molbev.a040454 3447015

[25] Bortolaia V, Kaas RS, Ruppe E, Roberts MC, Schwarz S, Cattoir V, et al. ResFinder 4.0 for predictions of phenotypes from genotypes. J Antimicrob Chemother. 2020;75: 3491–3500. doi: 10.1093/jac/dkaa345 32780112 PMC7662176

[26] Florensa AF, Kaas RS, Clausen PTLC, Aytan-Aktug D, Aarestrup FM. ResFinder-an open online resource for identification of antimicrobial resistance genes in next-generation sequencing data and prediction of phenotypes from genotypes. 2022; doi: 10.1099/mgen.0.000748 35072601 PMC8914360

[27] Zankari E, Allesøe R, Joensen KG, Cavaco LM, Lund O, Aarestrup FM. PointFinder: A novel web tool for WGS-based detection of antimicrobial resistance associated with chromosomal point mutations in bacterial pathogens. J Antimicrob Chemother. 2017;72: 2764–2768. doi: 10.1093/jac/dkx217 29091202 PMC5890747

[28] Liu B, Zheng D, Jin Q, Chen L, Yang J. VFDB 2019: a comparative pathogenomic platform with an interactive web interface. Nucleic Acids Res. 2019;47: D687–D692. doi: 10.1093/nar/gky1080 30395255 PMC6324032

[29] Robertson J, Nash JHE. MOB-suite: software tools for clustering, reconstruction and typing of plasmids from draft assemblies. Microb Genomics. 2018;4. doi: 10.1099/mgen.0.000206 30052170 PMC6159552

[30] Robertson J, Bessonov K, Schonfeld J, Nash JHE. Universal whole-sequence-based plasmid typing and its utility to prediction of host range and epidemiological surveillance. Microb Genomics. 2020;6: 1–12. doi: 10.1099/mgen.0.000435 32969786 PMC7660255

[31] Robertson J, Yoshida C, Kruczkiewicz P, Nadon C, Nichani A, Taboada EN, et al. Comprehensive assessment of the quality of *Salmonella* whole genome sequence data available in public sequence databases using the *Salmonella* in silico Typing Resource (SISTR). Microb genomics. 2018;4. doi: 10.1099/mgen.0.000151 29338812 PMC5857378

[32] Turcotte MR, Smith JT, Li J, Zhang X, Wolfe KL, Gao F, et al. Genome characteristics of clinical *Salmonella enterica* population from a state public health laboratory, New Hampshire, USA, 2017–2020. BMC Genomics. 2022;23: 1–11. doi: 10.1186/s12864-022-08769-1 35870884 PMC9308939

[33] Ibrahim GM, Morin PM. *Salmonella* serotyping using whole genome sequencing. Front Microbiol. 2018;9: 1–8. doi: 10.3389/fmicb.2018.02993 30619114 PMC6300517

[34] Banerji S, Simon S, Tille A, Fruth A, Flieger A. Genome-based Salmonella serotyping as the new gold standard. Sci Rep. 2020;10: 1–10. doi: 10.1038/s41598-020-61254-1 32152449 PMC7062728

[35] Worley J, Meng J, Allard MW, Brown EW, Timme RE. *Salmonella enterica* phylogeny based on whole-genome sequencing reveals two new clades and novel patterns of horizontally acquired genetic elements. MBio. 2018;9. doi: 10.1128/mBio.02303-18 30482836 PMC6282209

[36] Chattaway MA, Dallman TJ, Larkin L, Nair S, McCormick J, Mikhail A, et al. The transformation of reference microbiology methods and surveillance for *Salmonella* with the use of whole genome sequencing in England and Wales. Front Public Heal. 2019;7: 317. doi: 10.3389/fpubh.2019.00317 31824904 PMC6881236

[37] Saltykova A, Wuyts V, Mattheus W, Bertrand S, Roosens NHC, Marchal K, et al. Comparison of SNP-based subtyping workflows for bacterial isolates using WGS data, applied to Salmonella enterica serotype Typhimurium and serotype 1,4,[5],12:i:-. Mossong J, editor. PLoS One. 2018;13: e0192504. doi: 10.1371/journal.pone.0192504 29408896 PMC5800660

[38] Timme RE, Strain E, Baugher JD, Davis S, Gonzalez-Escalona N, Leon MS, et al. Phylogenomic pipeline validation for foodborne pathogen disease surveillance. J Clin Microbiol. 2019;57: e01816–18. doi: 10.1128/JCM.01816-18 30728194 PMC6498022

[39] Stasiewicz MJ, Oliver HF, Wiedmann M, den Bakker HC. Whole-genome sequencing allows for improved identification of persistent *Listeria monocytogenes* in food-associated environments. Appl Environ Microbiol. 2015;81: 6024–37. doi: 10.1128/AEM.01049-15 26116683 PMC4551262

[40] Register Federal. Almonds grown in California; outgoing quality control requirements. 7 CFR part 981. Fed Regist. 2007;72: 15021–15036. Available from: https://www.federalregister.gov/documents/2007/03/30/07-1557/almonds-grown-in-california-outgoing-quality-control-requirements

[41] Haendiges J, Davidson GR, Pettengill JB, Reed E, Ramachandran P, Blessington T, et al. Genomic evidence of environmental and resident *Salmonella* Senftenberg and Montevideo contamination in the pistachio supply-chain. PLoS One. 2021;16: e0259471. doi: 10.1371/JOURNAL.PONE.0259471 34735518 PMC8568146

[42] Russo ET, Biggerstaff G, Hoekstra RM, Meyer S, Patel N, Miller B, et al. A recurrent, multistate outbreak of *Salmonella* serotype Agona infections associated with dry, unsweetened cereal consumption, United States, 2008. J Food Prot. 2013;76: 227–230. doi: 10.4315/0362-028X.JFP-12-209 23433369

[43] Harris LJ, Lieberman V, Mashiana RP, Atwill E, Yang M, Chandler JC, et al. Prevalence and amounts of *Salmonella* found on raw California inshell pistachios. J Food Prot. 2016;79: 1304–1315. doi: 10.4315/0362-028X.JFP-16-054 27497117

[44] Carattoli A. Plasmids and the spread of resistance. Int J Med Microbiol. 2013;303: 298–304. doi: 10.1016/j.ijmm.2013.02.001 23499304

[45] Ondov BD, Treangen TJ, Melsted P, Mallonee AB, Bergman NH, Koren S, et al. Mash: Fast genome and metagenome distance estimation using MinHash. Genome Biol. 2016;17: 1–14. doi: 10.1186/s13059-016-0997-x 27323842 PMC4915045

[46] Haendiges J, Keller S, Suehr Q, Anderson N, Reed E, Zheng J, et al. Complete genome sequences of five *Salmonella enterica* strains used in knoculation cocktails in low-moisture food storage studies. Microbiol Resour Announc. 2019;8: e01588–18. 30643905 10.1128/MRA.01588-18 PMC6328678

[47] Schonfeld J, Clark C, Robertson J, Arya G, Eagle SHC, Gurnik S, et al. Complete Genome Sequences for 36 Canadian Salmonella enterica serovar Typhimurium and I 1,4,[5],12:i:–isolates from clinical and animal sources. Microbiol Resour Announc. 2021;10: 9–11. doi:10.1128/mra.00734-20 10.1128/MRA.00734-20 PMC8407683 33414281

[48] Villa L, García-Fernández A, Fortini D, Carattoli A. Replicon sequence typing of IncF plasmids carrying virulence and resistance determinants. J Antimicrob Chemother. 2010;65: 2518–2529. doi: 10.1093/jac/dkq347 20935300

[49] McMillan EA, Jackson CR, Frye JG. Transferable Plasmids of Salmonella enterica associated with antibiotic resistance genes. Front Microbiol. 2020;11. doi: 10.3389/fmicb.2020.562181 33133037 PMC7578388

[50] Nguyen S V., Harhay DM, Bono JL, Smith TPL, Fields PI, Dinsmore BA, et al. Complete and closed genome sequences of 10 Salmonella enterica subsp. enterica serovar Anatum isolates from human and bovine sources. Genome Announc. 2016;4: 3–4. doi: 10.1128/genomeA.00447-16 27257192 PMC4891638

[51] Caza M, Lépine F, Milot S, Dozois CM. Specific roles of the iroBCDEN genes in virulence of an avian pathogenic *Escherichia coli* O78 strain and in production of salmochelins. Infect Immun. 2008;76: 3539–3549. doi: 10.1128/IAI.00455-08 18541653 PMC2493193

[52] Li C, Pan D, Li M, Wang Y, Song L, Yu D, et al. Aerobactin-mediated iron acquisition enhances biofilm formation, oxidative stress resistance, and virulence of *Yersinia pseudotuberculosis*. Front Microbiol. 2021;12: 1–14. doi: 10.3389/fmicb.2021.699913 34335534 PMC8319957

[53] Fricke WF, McDermott PF, Mammel MK, Zhao S, Johnson TJ, Rasko DA, et al. Antimicrobial resistance-conferring plasmids with similarity to virulence plasmids from avian pathogenic *Escherichia coli* strains in *Salmonella enterica* serovar Kentucky isolates from poultry. Appl Environ Microbiol. 2009;75: 5963–5971. doi: 10.1128/AEM.00786-09 19648374 PMC2747853

[54] Tasmin R, Hasan NA, Grim CJ, Grant A, Choi SY, Alam MS, et al. Genotypic and phenotypic characterization of multidrug resistant *Salmonella Typhimurium* and Salmonella Kentucky strains recovered from chicken carcasses. Cloeckaert A, editor. PLoS One. 2017;12: e0176938. doi: 10.1371/journal.pone.0176938 28481935 PMC5421757

[55] Neuert S, Nair S, Day MR, Doumith M, Ashton PM, Mellor KC, et al. Prediction of phenotypic antimicrobial resistance profiles from whole genome sequences of non-typhoidal *Salmonella enterica*. Front Microbiol. 2018;9: 592. doi: 10.3389/fmicb.2018.00592 29636749 PMC5880904

[56] Hurtado R, Barh D, Weimer BC, Viana MVC, Profeta R, Sousa TJ, et al. WGS-Based lineage and antimicrobial resistance pattern of *Salmonella* Typhimurium isolated during 2000–2017 in Peru. Antibiotics. 2022;11: 1170. doi: 10.3390/ANTIBIOTICS11091170/S1 36139949 PMC9495214

[57] Tang B, Elbediwi M, Nambiar RB, Yang H, Lin J, Yue M. Genomic characterization of antimicrobial-resistant *Salmonella enterica* in duck, chicken, and pig farms and retail markets in Eastern China. Microbiol Spectr. 2022;10. doi: 10.1128/spectrum.01257-22 36047803 PMC9603869

[58] Rehman MA, Yin X, Persaud-Lachhman MG, Diarra MS. First detection of a fosfomycin resistance gene,fosA7, in *Salmonella enterica* serovar Heidelberg isolated from broiler chickens. Antimicrob Agents Chemother. 2017;61. doi: 10.1128/AAC.00410-17 28533247 PMC5527569

[59] McDermott PF, Tyson GH, Kabera C, Chen Y, Li C, Folster JP, et al. Whole-genome sequencing for detecting antimicrobial resistance in nontyphoidal *Salmonella*. Antimicrob Agents Chemother. 2016;60: 5515–20. doi: 10.1128/AAC.01030-16 27381390 PMC4997858

[60] Cheng RA, Eade CR, Wiedmann M. Embracing Diversity: Differences in virulence mechanisms, disease severity, and host adaptations contribute to the success of nontyphoidal Salmonella as a foodborne pathogen. Front Microbiol. 2019;10: 1–20. doi: 10.3389/fmicb.2019.01368 31316476 PMC6611429

[61] Hurtado-Escobar GA, Grépinet O, Raymond P, Abed N, Velge P, Virlogeux-Payant I. H-NS is the major repressor of *Salmonella* Typhimurium Pef fimbriae expression. Virulence. 2019;10: 849–867. doi: 10.1080/21505594.2019.1682752 31661351 PMC6844306

[62] Libby SJ, Adams LG, Ficht TA, Allen C, Whitford HA, Buchmeier NA, et al. The spv genes on the *Salmonella* Dublin virulence plasmid are required for severe enteritis and systemic infection in the natural host. Infect Immun. 1997;65: 1786–1792. doi: 10.1128/iai.65.5.1786-1792.1997 9125562 PMC175217

[63] Guiney DG, Fierer J. The role of the spv genes in *Salmonella* pathogenesis. Front Microbiol. 2011;2: 1–10. doi: 10.3389/fmicb.2011.00129 21716657 PMC3117207

[64] Krukonis ES, Thomson JJ. Complement evasion mechanisms of the systemic pathogens Yersiniae and Salmonellae. FEBS Lett. 2020;594: 2598–2620. doi: 10.1002/1873-3468.13771 32170725

